# *Sex Determining Region Y-Box 2 (SOX2)* Amplification Is an Independent Indicator of Disease Recurrence in Sinonasal Cancer

**DOI:** 10.1371/journal.pone.0059201

**Published:** 2013-03-27

**Authors:** Andreas Schröck, Friederike Göke, Patrick Wagner, Maike Bode, Alina Franzen, Martin Braun, Sebastian Huss, Abbas Agaimy, Stephan Ihrler, Ropika Menon, Robert Kirsten, Glen Kristiansen, Friedrich Bootz, Claudia Lengerke, Sven Perner

**Affiliations:** 1 Department of Otorhinolaryngology/Head and Neck Surgery, University of Bonn, Bonn, Germany; 2 Department of Prostate Cancer Research, University of Bonn, Bonn, Germany; 3 Institute of Pathology, University of Bonn, Bonn, Germany; 4 Winchester Medical Center, Winchester, Virginia, United States of America; 5 Institute of Pathology, University of Cologne, Cologne, Germany; 6 Institute of Pathology, University of Erlangen, Erlangen, Germany; 7 Laboratory for Dermatohistology and Oral Pathology; Institute of Pathology, Ludwig Maximilian University of Munich, Munich, Germany; 8 Medical Center II, University of Tuebingen, Tuebingen, Germany; Virginia Commonwealth University, United States of America

## Abstract

**Objectives:**

The transcription factor *SOX2* (3q26.3-q27) is an embryonic stem cell factor contributing to the induction of pluripotency in terminally differentiated somatic cells. Recently, amplification of the *SOX2* gene locus has been described in squamous cell carcinoma (SCC) of different organ sites. Aim of this study was to investigate amplification and expression status of *SOX2* in sinonasal carcinomas and to correlate the results with clinico-pathological data.

**Materials and Methods:**

A total of 119 primary tumor samples from the sinonasal region were assessed by fluorescence in-situ hybridization and immunohistochemistry for *SOX2* gene amplification and protein expression, respectively. Of these, 59 were SSCs, 18 sinonasal undifferentiated carcinomas (SNUC), 10 carcinomas associated with an inverted papilloma (INVC), 19 adenocarcinomas (AD) and 13 adenoid cystic carcinomas (ACC).

**Results:**

*SOX2* amplifications were found in subsets of SCCs (37.5%), SNUCs (35.3%), INVCs (37.5%) and ADs (8.3%) but not in ACCs. SOX2 amplification resulted in increased protein expression. Patients with *SOX2*-amplified sinonasal carcinomas showed a significantly higher rate of tumor recurrences than *SOX2* non-amplified tumors.

**Conclusion:**

This is the first study assessing *SOX2* amplification and expression in a large cohort of sinonasal carcinomas. As opposed to AD and ACC, *SOX2* amplifications were detected in more than 1/3 of all SCCs, SNUCs and INVCs. We therefore suggest that SNUCs are molecularly closely related to SCCs and INVCs and that these entities represent a subgroup of sinonasal carcinomas relying on SOX2 acquisition during oncogenesis. *SOX2* amplification appears to identify sinonasal carcinomas that are more likely to relapse after primary therapy, suggesting that these patients might benefit from a more aggressive therapy regime.

## Introduction

Recent advances in genetic profiling have led to more refined molecular classifications of specific tumor entities, providing novel diagnostic, prognostic and predictive biomarkers and paving the way for rational therapy regimens. However, the genetic landscape of rare tumor entities remains largely unelucidated. Malignant tumors of the paranasal sinuses or the nasal cavity account for less than 1% of all cancers and for about 3% of all malignant otorhinolaryngeal tumors [1]. The annual incidence rate is 0.5 to 1.0 per 100 000 people [2]. These tumors mainly occur within the maxillary sinus (∼60%) or the nasal cavity (∼30%) [1]. The most frequent histological entity is squamous cell carcinoma (SCC). The majority of patients presents at an advanced stage of disease due to a lack of early disease symptoms [3]. If at all feasible, complete resection of these mostly locally advanced tumors often results in severe cosmetic and functional compromise. Despite improvements in the field of surgery and radiochemotherapy, most patients suffering from carcinomas of the sinonasal origin have an unfavourable prognosis even in the setting of aggressive therapy [3], [4]. While controversy persists in determining the optimal therapeutic approach in a given patient, prognostic markers identifying patients who will benefit from more aggressive treatment and novel molecular targeted therapies are urgently needed.

The *SOX2* (SRY (sex determining region Y)-box 2) gene is located at the chromosomal locus 3q26.33 and encodes for a transcription factor containing the high mobility group (HMG) DNA-binding domain. *SOX2* is essential for maintenance of the pluripotency of embryonic stem cells and self-renewal of tissue-specific adult stem cells [5], [6], [7]. When co-expressed with other embryonic stem cell factors like *NANOG* and *Oct3/4*, *SOX2* is able to re-induce pluripotency in terminally differentiated cells [8], [9], [10]. More recently, deregulated SOX2 expression was noted in a variety of tumors [11], [12] suggesting that *SOX2* also plays important roles as an oncogene. *SOX2* amplification was first detected in lung SCCs with reported frequencies varying from 20% to 60% [13], [14], [15], [16], [17]. At much lower frequencies (6%) *SOX2* amplification has also been identified in adenocarcinoma of the lung [15], [16], [17], [18]. Apart from lung, aberration of this chromosomal region has been described in SCC of the esophagus [13], skin, cervix, and penis [12]. In the head and neck region, Freier et al. found recurrent *SOX2* amplification in 52% of oral SCCs [19].

To our knowledge, currently there is no data describing the role of SOX2 in carcinomas of the sinonasal region. We assembled a clinically well-characterized cohort comprising the most common sinonasal carcinomas, namely SCC, sinonasal undifferentiated carcinoma (SNUC), carcinoma associated with an inverted papilloma (INVC), adenocarcinoma (AD) and adenoid cystic carcinoma (ACC) and assessed for SOX2 amplification and protein expression status. Furthermore, we evaluated the prognostic impact of *SOX2* amplification and SOX2 protein expression levels by correlating these results with clinico-pathological data.

## Materials and Methods

### Cohort Characterization and Clinico-pathological Data Collection

A total of 119 patients with sinonasal carcinomas deriving from independent cohorts (University of Bonn (n = 87), University of Cologne (n = 27), Ludwig Maximilian University of Munich (n = 3) and University of Erlangen (n = 2)) were included in our study.

In detail, the Bonn cohort consisted of 38 sinonasal SCCs with 7 corresponding regional lymph node metastases, 13 SNUCs with 1 corresponding lymph node metastasis, 10 INVCs with 2 corresponding lymph node metastases, 13 ADs, and 13 ACCs, as well as 35 samples of normal sinonasal respiratory tissue. 21 sinonasal SCCs, and 6 ADs were retrieved from the University of Cologne. 3 and 2 SNUCs were retrieved from the University of Munich and Erlangen, respectively. By employing NUT-antibody (NUT C52B1, Cell Signaling Technology, Beverly, MA, USA) via immunohistochemistry, two NUT-midline carcinomas (NMC) previously misdiagnosed as SNUCs have been excluded from our cohort.

All patients were treated at the head and neck departments of the listed University Hospitals. Clinico-pathological data, including follow-up, were obtained via review of the patients' medical charts, which were available only for the Bonn cohort. The mean duration of follow-up was 33±37 months.

The study was approved by the internal review board of the University Hospital of Bonn (#267/11). Due to the fact that we used anonymized patients' FFPE material in a retrospective manner and the patients are no longer being treated at the clinic our internal review board disclaimed a written or verbal patients' consent to participate in this study.

### Tissue Microarray (TMA) Construction

Formalin-fixed, paraffin-embedded samples were cut into 4-µm thick sections, mounted on slides, and stained with hematoxylin and eosin. Histology was confirmed by two pathologists (S.P. and F.G.) to reassure diagnosis and to mark carcinoma tissue as target areas for TMA construction. TMA recipient blocks were constructed using a semiautomatic tissue microarray instrument (Beecher Instruments, Sun Prairie, WI). Three representative 0.6 mm cores of viable tissue from each tumor, its corresponding lymph node metastasis and benign tissue were included.

### Fluorescence In-situ Hybridization Assay (FISH)

*SOX2* amplification status was assessed on TMAs. For this purpose we performed FISH assays as described previously [13]. In brief, the *SOX2* target probe (red fluorescent signal) probe spanning the locus 3q26.33 (BAC clone CTD-2348H10, Invitrogen, Carlsbad, CA, USA) and a commercially available centromeric probe on chromosome 3 (Metasystems, Altlussheim, Germany) were selected for hybridisation. Only nuclei displaying green reference signals were included for the determination of the *SOX2* copy number status. All TMA slides were analyzed by two independent evaluators (A.S. and F.G.) under a 63x oil immersion objective with a fluorescence microscope (Zeiss, Jena, Germany). In each case, we assessed at least 100 tumor cell nuclei. A wild-type (WT) nucleus displayed the same amount of red and green signals in a cell. A sample was considered amplified if at least 30% nuclei displayed the *SOX2* amplification. Amplification status was defined according to the criteria of Wilbertz et al. [17]. In detail, a low level amplification (LLA) was defined as additional 2–9 red target signals exceeding the number of green signals. Additional ten or more red target signals or clusters of red target gene signals as compared to the green reference signals were defined as high-level amplification (HLA).

### Immunohistochemistry (IHC)

Immunohistochemistry was performed with the Ventana Discovery automated immunostaining system (Ventana Medical Systems, Tucson, AZ, USA), using Ventana reagents. Paraffin sections (5 µm) were mounted on superfrost slides, deparaffinised in anorganic buffer and then pretreated with EDTA-based buffer (pH 8.4). In order to assess protein expression status the primary antibody (polyclonal goat anti–human *SOX2* antibody, AF2018; R&D Systems, Minneapolis, MN; dilution, 1∶40, heat-induced epitope retrieval) was applied. Dilution was achieved with a Ventana diluent. The bound antibody was visualized using a biotinylated detection kit (diaminobenzidine and horseradish peroxidase (DABMap-kit, Ventana)). Counterstaining was performed with hematoxylin and Blueing Reagent (Ventana). Sections were washed, dehydrated in an ascending alcohol series and covered using Cytoseal. TMAs were digitalized using the Zeiss MIRAX DESK scanner. We then used a semi-automated quantitative image analysis software (Definiens Architect XD 1.2, Definiens, Munich, Germany) in order to obtain a continuous spectrum of average nuclear brown staining intensity in arbitrary units (maximum range of readout 0–200) for the tumor areas of each core.

### Statistics

For ordinally scaled nonparametric data, the Wilcoxon-Mann-Whitney-U Test was used. In case of more than two groups, we used the extended Wilcoxon-Mann-Whitney-U Test. Fisher’s exact test was used for computing statistical significance. Comparison of mean values was done by t-test or analysis of variance in case of more than two groups. Two groups according to the median value were defined for age (66 years). P values of p≤0.05 were considered to be statistically significant. All statistical analysis and graphical output were done with R version 2.13.0 on MAC OS X 10.6.8 system.

## Results

### *SOX2* Amplification Status (FISH)

**Figure 1:**
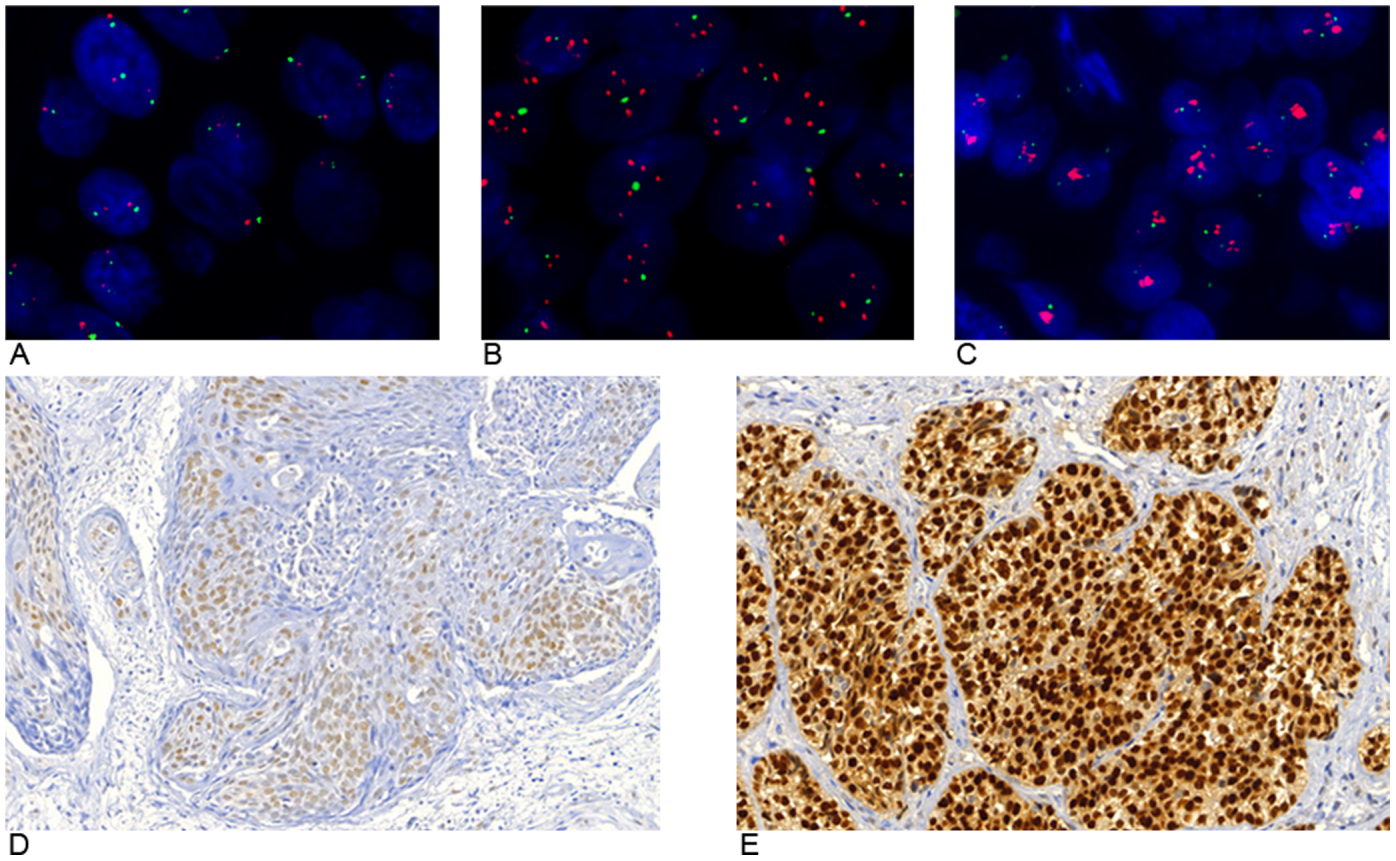
SOX2 FISH and SOX2 IHC. Above: representative FISH images of sinonasal squamous cell carcinoma samples without *SOX2* amplification (A), *SOX2* low level amplification (B) and *SOX2* high level amplification (C). Below: representative immunohistochemical stains of weak nuclear SOX2 expression in case of wildtype *SOX2* (D) and strong nuclear SOX2 expression in case harbouring *SOX2* amplification (E).

Of the 119 tumor samples 97 were assessable and 22 samples were not assessable due to technical reasons. Of assessable SCCs (48/59), we found wild-type *SOX2* in 62.5% (30/48), LLA in 35.4% (17/48) and HLA in 2.1% (1/48). Examples of each SOX2 copy number status are illustrated in **Figure 1**. Of all assessable SNUCs (17/18), 64.7% (11/17) displayed wild-type *SOX2*, 29.4% (5/17) LLA and 5.9% (1/17) HLA. Of assessable ADs (13/19) wild-type SOX2 was found in 92.3% (12/13) and LLA in 7.7% (1/13). INVCs (8/10 assessable) displayed wild-type SOX2 in 62.5% (5/8) and LLA in 37.5% (3/8). All assessable ACCs (11/13) uniformly showed wild-type *SOX2*. None of the 35 samples of normal respiratory tissue revealed evidence of *SOX2* amplification. Results are summarized in **Table 1**.

**Table 1:** *SOX2* amplification status in sinonasal carcinomas.

	Investigated Cases	Assessable Cases	LLA	HLA	WT
SCC	59	48	17 (35.4%)	1 (2.1%)	30 (62.5%)
SNUC	18	17	5 (29.4%)	1 (5.9%)	11 (64.7%)
AD	19	13	1 (7.7%)	0	12 (92.3%)
INVC	10	8	3 (37.5%)	0	5 (62.5%)
ADC	13	11	0	0	11 (100%)
Total	119	97	26 (26.8%)	2 (2.1%)	69 (71.1%)

LLA: *SOX2* low level amplification; HLA: *SOX2* high level amplification; WT: *SOX2* wildtype; SCC: squamous cell carcinoma; SNUC: sinonasal undifferentiated carcinoma; AD: adenocarcinoma; INVC: carcinoma out of an inverted sinonasal papilloma; ACC: adenoid cystic carcinoma.

### SOX2 Protein Expression Levels (IHC)

**Figure 2:**
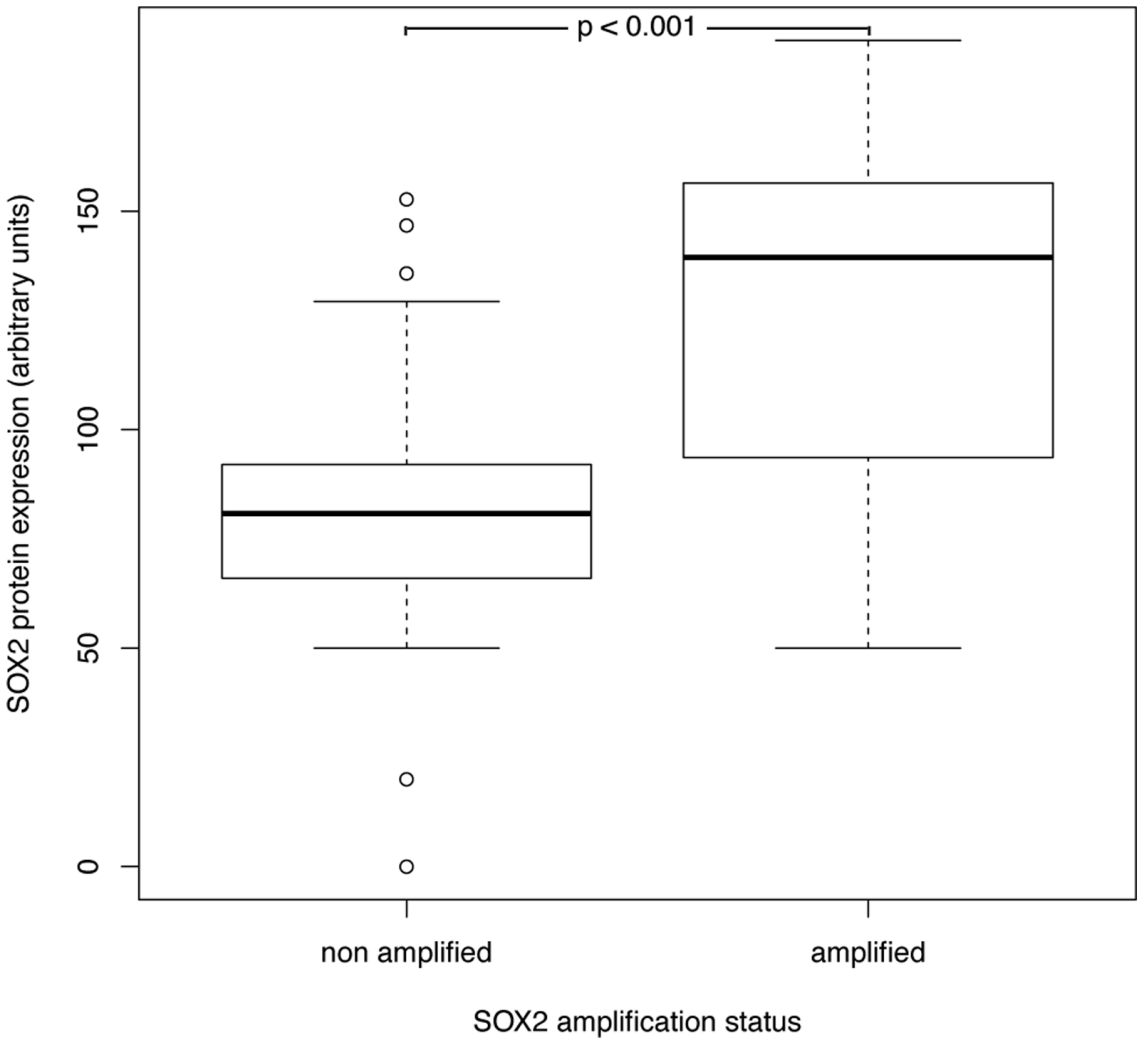
SOX2 protein expression levels. *SOX2-*amplified sinonasal tumors exhibited significantly higher SOX protein expression than non-amplified tumors.

SOX2 protein expression was highly heterogenous among the analyzed cohort samples, resulting in values from 0 to 189 (arbitrary units). Comparing the different histologies, SCC exhibited the highest protein expression (102±40) followed by INVC (86±27), SNUC (82±46), AD (45±30) and ACC (38±30). Mean SOX2 expression in SCC, INVC and SNUC was significantly higher in SOX2 amplified cancer samples (125±40) than in non-amplified tumors (80±33) (p<0.001) (**Figure 1** and **2**). Interestingly, 14% of assessable non-amplified samples also displayed a high SOX2 protein expression (more than the median protein expression of amplified tumors) indicating that SOX2 activation can occur through alternative mechanisms.

### *SOX2* Amplification and Expression in Metastatic Lymph Nodes in Sinonasal SCC, INVC and SNUC

**Figure 3:**
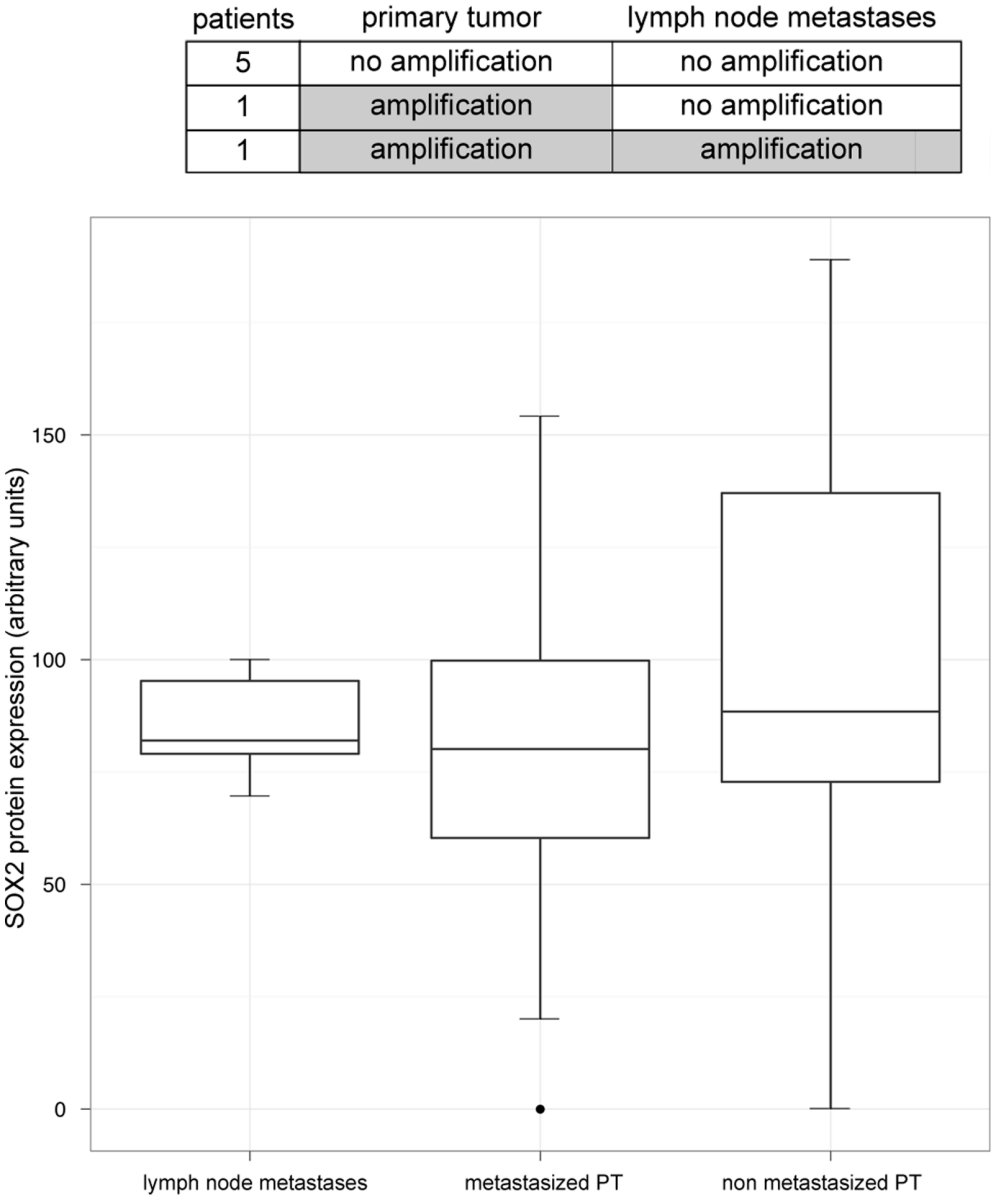
*SOX2* amplification status and protein expression in primary tumors and their corresponding lymph node metastases. Above, *SOX2* amplification status of primary sinonasal tumors and corresponding lymph node metastases. In most cases, *SOX2* gene copy number status is transferred into corresponding lymph node metastases. Below, we demonstrate SOX2 protein expression levels of lymph node metastases, their corresponding metastasized primary tumors (PT) and non metastasized primary tumors (PT). SOX2 protein expression level is not an independent predictor for the occurrence of lymph node metastases.

Furthermore, we analysed *SOX2* amplification and expression in primary sinonasal SCCs, INVs and SNUCs and their corresponding metastatic lymph nodes of the Bonn cohort (n = 61). Information on the lymph node status was available in 54 out of 61 patients. Of these, 10 displayed regional lymph node metastases. 7/10 samples of lymph node metastases were assessable. 5 out of these 7 (71%) patients did not display *SOX2* amplification either in the primary tumor or in the metastasis (**Figure 3**). One patient harboured a SOX2 LLA in the primary tumor but not in the lymph node metastasis. One patient with *SOX2* LLA in the primary tumor also exhibited a LLA in the corresponding lymph node metastasis. No differences in *SOX2* protein expression were noted across these samples (**Figure 3**).

### Prognostic Value of *SOX2* Amplification/Expression

**Figure 4:**
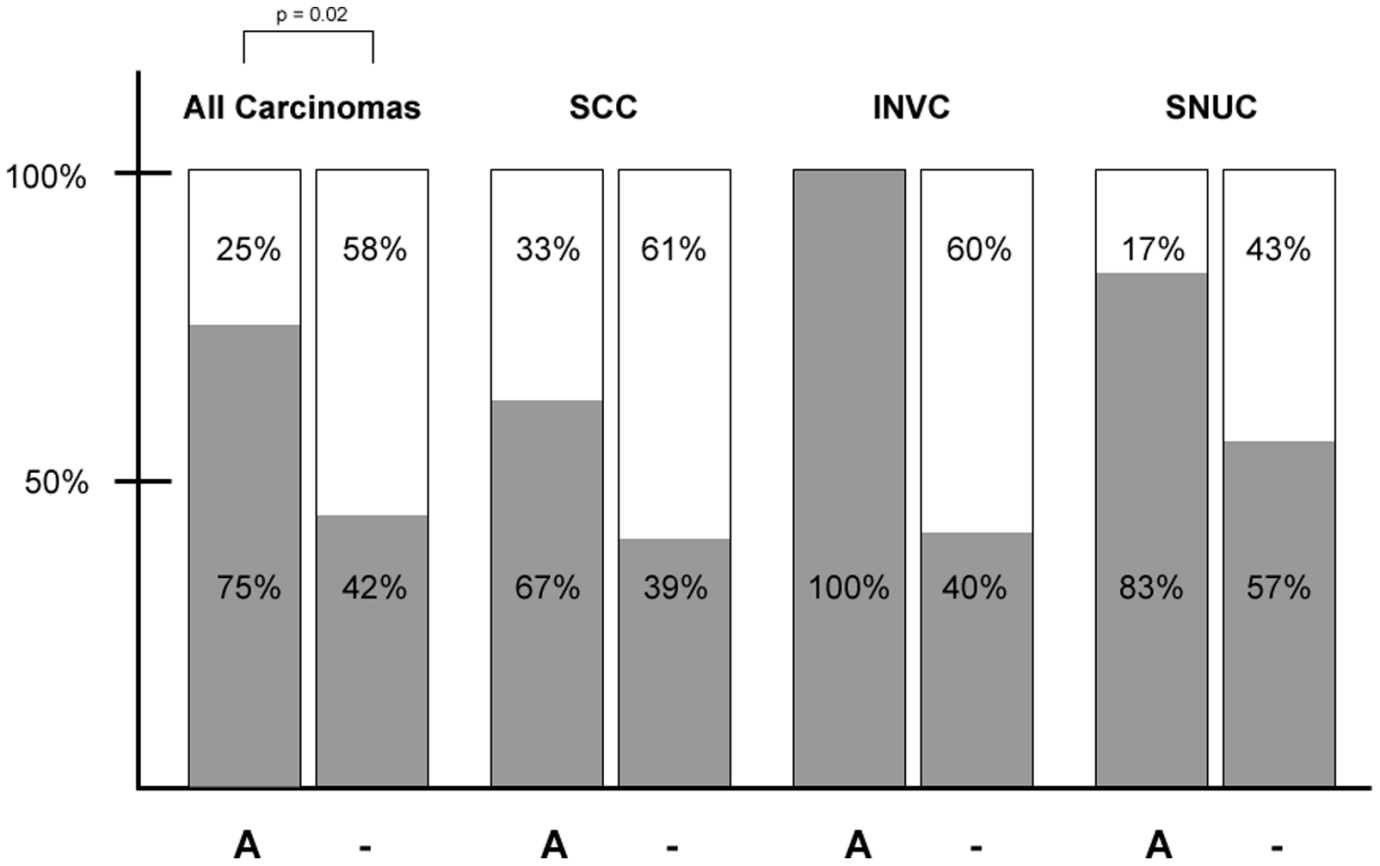
Tumor recurrence in relation to the *SOX2* amplification status. Incidence of tumor recurrence (red bars) versus relapse-free survival (white bars) of SOX2-amplified (A) and non-amplified (−) tumors subdivided into the different tumor histologies. Due to the low number of patients, p-value was calculated for all tumor entities together (SCC: squamous cell carcinoma; INVC: carcinoma out of an inverted sinonasal papilloma SNUC: sinonasal undifferentiated carcinoma).

In order to assess the prognostic value of *SOX2* amplification on the clinical course of disease in sinonasal SCCs, INVCs and SNUCs, we correlated the *SOX2* amplification status with the incidence of tumor recurrence. Irrespective of the specific histology, patients with *SOX2*-amplified SCCs, SNUCs, and INVCs experienced a significantly higher incidence of recurrence (15/20; 75%) as opposed to non-amplified carcinomas (13/31; 42%) (p = 0.02) (**Figure 4**). However, we could not detect a significant correlation between SOX2 protein expression levels and tumor recurrence. In tendency, the overall survival rate after 3 years was lower for *SOX2*-amplified patients as compared to *SOX2* non-amplified patients (25.7% versus 58.6%). However, this did not reach statistical significance (p = 0.28).

### Correlation of *SOX2* Amplification/Expression Status with Clinico-pathological Data

Within the group of patients suffering from SCC, INVC and SNUC, age and gender were similar in patients with *SOX2* wild-type or amplified tumors. No association between *SOX2* amplification status and patient’s smoking status, tumor differentiation or stage was noted (**Table 2**). SOX2 protein expression status did not correlate with age, gender, smoking status, tumor differentiation or stage.

**Table 2:** Association of clinico-pathological data with *SOX2* amplification status.

	*SOX2* Amplification	*SOX2* Wild-Type	p-value
Gender			0.50
male	13 (35.1%)	24 (64.9%)	
female	8 (44.4%)	10 (55.6%)	
Age, median			0.58
<66	13 (40.6%)	19 (59.4%)	
≥ 66	7 (31.8%)	15 (68.2%)	
smoking habits			0.72
smoker	8 (42.1%)	11 (57.9%)	
non-smoker	5 (31.2%)	11 (68.6%)	
Overall survival			0.28
1 year survival	65%	70%	
2 year survival	60%	63%	
3 year survival	26%	59%	
Tumor stage			0.39
T1	2 (22.2%)	7 (77.8%)	
T2	6 (50%)	6 (50%)	
T3	2 (22.2%)	7 (77.8%)	
T4	11 (44%)	14 (56%)	
Tumor differentiation			0.17
well	1 (16.6%)	5 (83.4%)	
moderate	9 (32.1%)	19 (67.9%)	
poor	4 (66.6%)	2 (33.4%)	

## Discussion

Deletion of tumor suppressor genes and/or amplification of oncogenes are frequently found in malignant tumors and play a critical role in tumorigenesis [20], [21]. Identification and characterization of these genetic pathways is necessary to understand tumor initiation, development and biology leading to the discovery of useful diagnostic, prognostic and predictive markers as well as potential targets for actionable therapies [21], [22]. The transcription factor *SOX2*, which is located at 3q26.33 controls cell self-renewal and differentiation processes in pluripotent stem cells and also during early embryonic development. Moreover, it has been identified as the driving oncogene of the 3q amplicon in SCCs of different organ sites, implying that *SOX2* is involved in SCC carcinogenesis [17] of different organ sites. In this study, we identified *SOX2* amplification as a novel event in carcinomas of the sinonasal region. 119 samples of sinonasal carcinomas were assessed in a semi-quantitative manner. Whereas *SOX2* amplification occurred in about 35% of SCCs, SNUCs and INVCs, it was a rare event in ADs and not detected in ACCs. To date, a definite histological identification and subcategorization of SNUCs as an independant tumor entity remains challenging. Interestingly, molecularbiological features such as SOX2 amplification and expression display striking similarities between undifferentiated SCCs and SNUCs. This may prove a close relationship between these two tumor entities, or even suggest that in fact SNUCs are dedifferentiated SCCs.

Since SOX2 protein expression levels were higher in SCCs, SNUCs and INVCs compared to ADs and ACCs regardless SOX2 gene copy number status, we hypothesize that SOX2 plays a more important role in these 3 tumor entities. Reflecting a gene-dosage effect, *SOX2* amplified carcinomas displayed a significantly higher SOX2 protein level than non-amplified carcinomas. However, we detected a high SOX2 protein expression in 14% of non-amplified carcinomas suggesting that in these cases the aberrant protein expression is driven by mechanisms other than gene amplification, as also previously reported by us in breast and ovarian cancers [11]. However, similar to SCC of the lung [17], our results imply that in sinonasal SCCs, INVCs and SNUCs increased *SOX2* gene copy numbers are the main driver of increasing SOX2 protein levels. There was a high correlation between SOX2 protein expression in primary tumors and lymph nodes metastases with all SOX2 expressing primary tumors also having SOX2 positive lymph node metastases. Mean SOX2 expression levels were comparable in metastatic versus primary tumor tissues. These data suggest that SOX2 expression is an early event during tumorigenesis rather than a genetic event acquired during tumor progression, similar to our observations in breast cancer where aberrant SOX2 expression was already observed in the earliest stages of tumor development [11]. One of the analyzed samples showed loss of SOX2 amplification in metastatic versus primary tumor tissue. This could be explained by clonal heterogeneity in this individual tumor with additional genetic events strongly driving metastasis in a SOX2-negative population. However, because of the very limited number of paired samples of primary tumor and corresponding metastatic lymph node, future studies on larger cohorts are needed to draw solid conclusions.

More recently, SOX2 and other embryonic proteins have been hypothesized to identify the tumor stem cell subpopulation within malignancies [14], [23], [24]. Tumor stem cells are thought to exhibit enhanced resistance to conventional anti-tumor therapies causing tumor recurrences and metastases [25]. In our study, SOX2 was homogenously expressed within the tumors. This indicates that SOX2 does not serve as a tumor stem cell marker in sinonasal carcinoma, but rather functions as a ubiquitously activated oncogene.

Concerning the prognostic value of SOX2 expression in tumors, contradictory results have been reported in different tumor entities, depending on their localization and histology: while some studies suggest that increased SOX2 expression is associated with a prolonged overall survival in patients suffering from lung SCCs [13], [17], [26] others refer to a high SOX2 expression as a marker of poor prognosis in esophageal SCC [27], oral SCC [28] or lung AD [18]. Although limited by a rather low number of cases, patients harbouring *SOX2* amplifications exhibited a significantly higher rate of tumor recurrences than patients lacking this event. This has two major implications. Firstly, SOX2 could serve as a valuable marker identifying patients with higher risk of disease recurrence. These patients could consecutively be introduced to closer follow-up strategies. Secondly, patients harbouring SOX2 amplification could profit from more aggressive primary therapy strategies, also reducing their risk of relapse.

We did not find a statistically significant association between *SOX2* amplification/expression status and the overall survival rate or prognostic factors such as tumor stage and regional lymph node status. Further studies performed on larger cohorts are needed to explore the value of *SOX2* as a prognostic marker in sinonasal carcinoma.

In summary, we identified *SOX2* as a novel oncogene expressed in a distinct subgroup of sinonasal SCCs, INVCs and SNUCs. SOX2 could serve as a valuable prognostic marker in these rare tumor entities.
